# The Role of Social Support and Belonging in Predicting Recovery from Problem Gambling

**DOI:** 10.1007/s10899-023-10225-y

**Published:** 2023-06-07

**Authors:** Katy L. Penfold, Jane Ogden

**Affiliations:** School of Psychology, University of Surrey, Guildford, GU2 7XH UK

**Keywords:** Gamblers anonymous, Gambling, Mutual aid, Peer support, COVID-19, Gambling addiction, Social support, Belonging

## Abstract

Research indicates a role for both social support and belonging in addiction recovery, however little is known about the role of these constructs in the recovery from problem gambling, and whether they relate to the effectiveness of mutual aid groups such as Gamblers Anonymous. The aim of this study was therefore to explore the relationship between social support and belonging, and to assess the role of demographics (including group membership of GA), social support and/or belongingness in predicting gambling addiction recovery in terms of gambling urges and quality of life. Using a cross sectional design, participants identifying as having problem gambling (n = 60) completed an online questionnaire with two independent variables (Social Support and Belonging), two dependent variables (Gambling Urges and Quality of Life) to assess gambling addiction recovery and measures of GA membership. The results showed no significant association between gender, age, ethnicity, education or employment status and gambling urges or quality of life. Membership to GA, and length of membership were significantly associated with gambling recovery indicating that being a member of GA and longer membership was associated with lower gambling urges and higher quality of life. Further, the results showed a high but not perfect correlation between social support and belonging (r(58) = .81, *p* =  < .01). A regression analysis showed that although there was a significant correlation between social support and belongingness, they played different roles in gambling addiction recovery. Social support alone predicted higher quality of life, but not a reduction in gambling urges; belonging (along with being a member of GA) predicted a reduction in gambling urges, but not an increase in quality of life. Social support and belonging have a differential impact on aspects of gambling addiction, and should be considered as different constructs. In particular, whilst the process underpinning reduced gambling urges is membership of GA and the sense of belonging it provides its members, social support per se is a better predictor of quality of life. These findings have implications for the development of treatment for problem gamblers in the future.

## Introduction

Problem gambling affects between 55 and 517 million people worldwide (Calado & Griffiths, 2016). In the UK alone, around half-a-million adults experience problem gambling, with a further two-and-a-half million people at low or moderate risk (Sullivan, 2019). People who experience problem gambling can face a range of complex and multifaceted difficulties depending on their personal circumstances which can include substantial financial loss, physical and mental decline, and breakdown in family relationships, and can affect both individuals and the societies in which they live (e.g. Battersby et al., 2006; Cunningham-Williams et al., 1998; Ferland et al., 2008; Dowling et al., 2009; Kalischuk et al., 2006).

Despite the number of people affected by problem gambling, there remains no clear pathway for recovery. This is partially because, as with other mental health issues, each situation can be incredibly complex with no “one size fits all” approach. It is also because little research has been carried out in this area, especially when compared to other addictions such as drugs or alcohol. Nonetheless, several treatment options have been identified. These include mutual aid groups, pharmacotherapies, family-marital therapies, psychoanalytic and psychodynamic approaches, behavioural therapy, cognitive therapy, cognitive-behavioural therapy and brief and motivational approaches (Petry, 2005). The most widely available and frequently accessed of these is the 12-step mutual aid fellowship Gamblers Anonymous.

Mutual aid groups such as Gamblers Anonymous are nonprofessional, self-supporting, and apolitical. They also tend to offer empathy and assistance rather than advice. Crucially, they are free and the only requirement for joining is a desire to be abstinent.

Alcoholics Anonymous (AA) is probably the most well-known (and well-researched) of the mutual aid groups (others include, for example, Narcotics Anonymous (NA), Eating Disorder Anonymous (EDA) and Sex Addict Anonymous (SAA)), although hundreds of “alcoholic mutual aid societies” offering peer-based support existed many years before the AA was founded in 1935 (White, 2001). Gamblers Anonymous is based on the principles of AA, but differs in that it places greater emphasis on assisting with matters associated with the financial side of gambling problems, and focuses more on the involvement of family members and social networks (Ferentzy et al., 2010).

The literature around mutual aid groups is mostly concerned with groups for alcohol or substance use (AA or NA), with research into groups for gambling (GA) only beginning to gain traction within the last few years. Furthermore, most of this research has been concerned with the clinical effectiveness of mutual aid groups (e.g. Kelly et al., 2020; Schuler et al., 2016); much less is known about the mechanisms underpinning them, though some studies have indicated that they work via several mechanisms simultaneously (Kelly et al., 2020; White et al., 2020). For example, Kelly and colleagues (2020) categorised the mechanisms of AA’s effectiveness along four lines: spirituality (e.g., prayer/meditation), common factors (therapeutic benefits provided by AA and also professional help services), AA-specific factors (such as working the steps or having a sponsor), and social factors (such as social support). Furthermore, White (2001) demonstrated that mutual aid groups have been leveraging aspects of social support such as social identity and social belonging to help their members stay sober since before the first 12-step groups were founded.

Social support refers to “support accessible to an individual through social ties to other individuals, groups, and the larger community” (Lin et al., 1979). This may be practical support such as doing chores, tangible support such as giving money, or emotional support. Forms of social support are the focus of the present study.

Groh and colleagues (2008) performed a systematic review in an attempt to gain some insight into the role social network variables play in AA, which showed an association between a range of social network variables including quality of friendships, greater friendship resources, greater social support, reduced support for alcohol consumption by friends, and increased support for abstaining from alcohol by friends, and involvement with AA. These results were further supported by qualitative findings which suggested that relationships made within AA provided individuals with more support, trust, and respect than relationships outside of AA, or those made prior to engagement with AA. Social support was also found to consistently mediate the effect of AA on abstinence, which suggests that the mechanism underpinning AA is social support.

Social support is linked to several health and wellbeing outcomes. Not only has social support been demonstrated to help maintain psychological wellbeing by reducing psychological stress, such as anxiety or depression, but has also been found to help adjustment to chronically stressful conditions such as coronary heart disease, HIV or stroke, to name a few (see Taylor, 2011 for review). Social support has also been demonstrated to play a significant role in both abstinence and recovery from various addictions (e.g. Best et al., 2016; Buckingham et al., 2013; Dingle et al., 2015).

Mutual aid groups may also provide their members with a sense of belonging. Belonging is a fundamental human need, and plays a crucial role in determining a range of health and wellbeing outcomes (see Allen et al., 2021 for review). This is especially the case for individuals who are going through disruptive life transitions, such as retirement, becoming a new mother, recovering from serious illness or changing from school to university (see Haslam et al., 2018 for reviews). Belonging to and identifying with a social group has also been demonstrated to reduce chances of experiencing depression (Sani et al., 2012), help ease symptoms of post-traumatic stress (Muldoon & Downes, 2007), and play a role in substance and alcohol addiction recovery (Dingle et al., 2015).

Though sometimes used interchangeably, social support and belonging differ; whilst social support refers to the provision of practical or emotional assistance to others from anyone in the person’s social network, belonging refers to “a unique and subjective experience that relates to a yearning for connection with others, the need for positive regard and the desire for interpersonal connection” (Rogers, 1951). In this sense, belonging is more about the individual’s perception of the quality of their relationships, rather than simply participation in them. Thus, it is entirely possible that an individual may receive extensive social support, but not achieve a sense of belonging, and vice versa.

The dearth of research into GA is surprising with just four studies being been published on GA in the UK in the past twenty-five years (all were in England, and all in the past four years;—Hutchison et al., 2018; Penfold & Ogden, 2021, 2022 under review; Rogers, 2019). Each of these studies highlight the social element of GA meetings. In particular, Rogers (2019) observed 20 “open” GA meetings in the UK, as well as interviewing 8 regular attendees to explore the process and content of GA meetings. Their results indicated that attending GA formed part of a crucial social network which was helpful in maintaining abstinence goals. Similarly, Hutchison et al. (2018) investigated the relationship between recovery group identification, social support received and provided to the group, and various recovery-related outcomes (such as abstinence self-efficacy and perceived risk in gambling ‘trigger’ situations) among people (n = 44) attending Gamblers Anonymous (GA). They found that identifying with a recovery group predicted more perceived support received from the group, more abstinence self-efficacy and less perceived risk in gambling-related ‘trigger’ situations. Furthermore, they also found that individuals which identified more with the recovery group also indicated that they provided more support to other members, which in turn was related to increased abstinence self-efficacy and less perceived risk in ‘trigger’ situations. Interestingly, the connections between recovery group identification and both recovery outcomes were found to be mediated by the perceived *provision* of social support, rather than its receipt. Likewise, Penfold and Ogden (2021) also explored the role of social support and interviewed 21 members of GA about their experiences of attending GA during the COVID-19 lockdown. During this time, members were forced to hold GA meetings online, via Zoom; something which the organisation had never done before. One of the main themes centered around social variables, with great importance being placed on the relationships made within GA. Members used the group for social comparison and social affirmation, as well as gaining a sense of solidarity with others. They also described creating strong and lasting social networks which were maintained by a sense of solidarity to the group. Crucially, they felt the group provided connection to others, with one participant directly stating “if addiction is loneliness, the opposite of addiction is connection”. A further study by Penfold & Ogden (2022, under review) supported these results; participants described a range of different social processes within the groups which they felt were important, including social comparison, and emotional and physical connection. Furthermore, the results suggest a key feature contributing to the success of GA is that the help came from other gamblers; the shared experience of gambling facilitated emotional connection to others in the group which in turn made them seem more trustworthy and legitimate than other professional service providers.

The results of these studies suggest that underpinning this sense of ‘connection’ is an intricate mixture of both social support and belonging, rather than one or the other. Whilst the benefits of both social support and belonging are known in addiction recovery, there has been no study to the researchers’ knowledge which directly compares the impact of both on gambling addiction, the extent to which either of these constructs predict gambling addiction recovery, and whether it is either of these mechanisms which contribute to the success of GA.

In summary, whilst research indicates that both social support and belonging are associated with addiction recovery these two constructs are often used interchangeably. Further, the mechanisms behind the effectiveness of mutual aid groups and the role of social support and belonging in promoting addiction recovery remains unclear. The purpose of this study therefore was to ascertain whether it is in fact social support, belonging to a group, or both, that predict addiction recovery in those with problem gambling. The specific research questions were ‘what is the relationship between social support and belonging in this group?’ and ‘to what extent do demographics (including group membership of GA), social support and/or belongingness predict gambling addiction recovery?

## Method

### Design

A cross-sectional questionnaire design was used, with two independent variables (Social Support and Belonging) and two dependent variables (Gambling Urges and Quality of Life) to assess gambling addiction recovery. Data was collected between April and May 2022, after the COVID-19 pandemic lockdown rules were eased in the UK; GA meetings were being carried out as a hybrid of online and face-to-face.

### Participants

A mixture of purposive and snowball sampling was used. Participants who had taken part in previous studies and had agreed to be contacted again were invited (via email) to take part. A link to the study was also posted on social media (Facebook and Instagram).

Participants (n = 60) were individuals aged 18 and over who felt they had experienced problems with their gambling. The study aimed to recruit a balanced number of individuals who either attend GA or not but this was not achieved, with the final sample including 38 GA members and 22 non-GA members. Most participants were male, White, aged 33–37, college/A-level educated, and most were employed. Of those who were members of Gamblers Anonymous, length of time as a member ranged from 1 day (they had requested to join on the day of participation) to 19 years.

### Measures

Participants completed the following measures. Reliability of scales was assessed using Cronbach’s alpha where appropriate.

#### Social Support

To measure social support, the Social Support Questionnaire short form (SSQ6; Sarason et al., 1987) was used. This is an empirically validated and widely used self-report measure of social support comprising 6 questions relating to social support satisfaction (i.e., “How satisfied are you that you have people who you can count on to be dependable when you need help?”). Questions are answered on a 6-point Likert scale ranging from “Very dissatisfied” to “Very satisfied”. A higher score reflects greater satisfaction (α = 0.98).

#### Belonging

To measure belonging, the General Belongingness Scale (Malone et al., 2012) was used. Malone and colleagues developed this scale in response to a lack of any other psychometrically sound way to measure belongingness prior. It is a 12-item measure with a 2-factor structure (Acceptance/Inclusion – 6 items—e.g. “when I am with other people I feel included”) and lack of Rejection/Exclusion – 6 items—e.g. “I feel like an outsider”) and has been demonstrated to have high reliability and strong patterns of validity (α = 0.97).

#### Gambling Addiction Recovery

Gambling addiction recovery was measured in two ways: quality of life and urge to gamble.

##### Quality of Life

Treatment outcomes in gambling addiction are not adequately defined and thus there are inconsistencies in how they are measured across studies (Pickering et al., 2018). This may be because there is no conceptually sound and widely agreed-upon definition of gambling addiction recovery, other than the absence of any diagnostic symptoms during a 12-month period (American Psychiatric Association, 2013). Furthermore, the course of gambling addiction can vary a great deal from one individual to another, which is reflected in the description of gambling disorder in the DSM-5 as either “episodic” or “persistent”. Because of this, a myriad of outcome measures are used in research relating to gambling addiction (Walker et al., 2006). As such, quality of life (QoL) is regularly used as this is an all-encompassing measure, which is broad enough to capture the myriad experiences of gambling disorder (Bonfils et al., 2019). Thus, a single-item quality of life measure (taken from the Australian Treatment Outcomes Profile; Deacon et al., 2020) was used to measure gambling addiction recovery. Participants were asked “How would you rate your overall quality of life in the past four weeks?” and answer on an 11-point measure anchored at 0 (poor) and 10 (good) (α = n/a).

##### Gambling Urges

In addition to quality of life, gambling urges were also measured. The justification for measuring gambling *urges* rather than gambling *behaviour* was that at least some of the sample were expected to be active members of Gamblers Anonymous and could therefore reasonably be assumed to have been abstinent from gambling at the time of data collection. Gambling urges were measured using the Gambling Urge Scale (Raylu & Oei, 2004b). This is a 6-item scale, answered on a 7-point semantic differential scale which has been demonstrated to have good reliability and validity (Raylu & Oei, 2004b; Smith et al., 2013) and has been used frequently in the gambling literature (α = 0.98).

##### Procedure

The recruitment email contained a link to the study, which was hosted on Qualtrics. Upon clicking the link, participants were presented with the information sheet, which detailed the study. Next, participants were presented with a consent form. Once informed consent was obtained, they were presented with a demographics form. It was made clear that giving this information was voluntary. They then proceeded to the study. All participants were asked to complete a set of questionnaires, comprising the SSQ6, the GBS, the GUS, the measure of QoL (see Appendix 6). The order in which these were presented was randomised to avoid order effects. Once complete, participants were presented with a debrief (containing relevant signposting) and thanked for their time.

## Results

### Data Analysis

Data was analysed in the following ways: (i) to describe the distribution of predictor and outcome variables; (ii) to describe participant demographics, predictor and outcome variables using descriptive statistics; (iii) to assess associations between social support, belonging and measures of gambling addiction recovery using univariate correlations; (iv) to assess the best predictors of gambling addiction recovery in terms of gambling urges and quality of life using multiple regression.

(i) Distribution of data

To assess the distribution of data, first the data were tested for normality by calculating the skewness and kurtosis of each variable. This data is presented in Table 1**.** From this it can be seen that all variables were non-parametric, therefore the appropriate non-parametric tests were used in the following analysis.

(ii) Participant demographics

**Table 1:** Distribution of data

Variable	Skewness	Kurtosis	Parametric/Non-parametric
Social support	− .516	− 1.200	Non-parametric
Belonging	− .350	− 1.169	Non-parametric
Quality of life	− .276	− 1.362	Non-parametric
Gambling urges	− .315	− 1.464	Non-parametric

Most participants were male, White, aged 33–37, college/A-level educated, and most were employed. Of those who were members of Gamblers Anonymous, length of time as a member ranged from 1 day (they had requested to join on the day of participation) to 19 years. This data is presented in Table 2.

(iii) Describing predictor and outcome variables

**Table 2:** Participant demographics

	All (*n* = *60)*
*Gender*	
Male	42 (70%)
Female	16 (26.7%)
Prefer not to say	1 (1.7%)
*Age*	
18–22	3 (5%)
23–27	3 (5%)
28–32	7 (11.7%)
33–37	19 (31.7%)
38–42	6 (10%)
43–47	5 (8.3%)
48–52	2 (3.3%)
53–57	3 (5%)
58–62	5 (8.3%)
63–72	1 (1.7%)
73–77	3 (5%)
*Ethnicity*	
Black	6 (10%)
Mixed race	1 (1.7%)
White	53 (88.3%)
*Level of education*	
Secondary school	7 (11.7%)
College/A level	24 (40%)
Undergraduate degree	16 (26.7%)
Postgraduate degree	10 (16.7%)
*Employment status*	
Employed	58 (96.6%)
Not employed	2 (3.3%)
*Member of GA*	
Yes	22 (36.7%)
No	38 (63.3%)
*Duration of membership to GA*	
> 1 year	1 (1.7%)
1–5 years	12 (20%)
6–10 years	3 (5%)
11–15 years	4 (6.7%)
16–20 years	2 (3.3%)

To provide a description of the data and for ease of comparison, each of the scales were recoded into “low”, “medium” and “high”. The majority of participants showed high social support, high belonging, high quality of life and low gambling urges. This data, along with means and standard deviations are presented in Table 3.

(iv) Associations between predictor and outcome variables

**Table 3:** Description of predictor and outcome variables

	*M* (SD)	Low	Medium	High
Social support	4.04 (1.77)	15 (25%)	19 (31.7%)	26 (43.3%)
Belonging	4.47 (2.17)	15 (25%)	18 (30%)	27 (45%)
Quality of life	5.95 (2.64)	20 (33.3%)	17 (28.3%)	23 (38.3%)
Gambling urges	3.32 (2.17)	35 (58.3%)	5 (8.3%)	20 (33.3%)

Prior to the multiple regression analysis, Spearman’s Rho univariate correlations were carried out to assess whether scores on the outcome variables were associated with any of the demographic characteristics. The results showed no significant association between gender, age, ethnicity, education or employment status and gambling urges or quality of life. Membership to GA, and length of membership were significantly associated with gambling recovery indicating that being a member of GA and longer membership was associated with lower gambling urges and higher quality of life. This data is presented in Table 4.

**Table 4:** Association between demographic characteristics and outcome variables

Demographic	Social support	Belonging	Gambling urges	Quality of life
Membership to GA	r(58) = .55, *p* = < .01	r(58) = .41, *p* = < .01	r(58) = .46, *p* = < .01	R(58) = .43, *p* = < .01
Duration of membership to GA	r(20) = .59, *p* = < .05	r(20) = .50, *p* = < .05	r(20) = − .56, *p* = < .05	r(20) = .48, *p* = < .05
Gender	r(57) = -.18, *p* = .18	r(57) = − .16, *p* = .24	r(57) = .17, *p* = .20	r(57) = -.06, *p* = .65
Age	r(57) = .25, *p* = .06	r(57) = .19, *p* = .15	r(57) = − .16, *p* = 24	r(57) = .01, *p* = .20
Ethnicity	r(55) = − .02, *p* = .86	r(57) = .01, *p* = .93	r(57) = .16, *p* = .25	r(57) = .04, *p* = .77
Education	r(55) = − .09, *p* = .52	r(57) = − .15, *p* = .25	r(57) = .01, *p* = .94	r(57) = .05, *p* = .72
Employment	r(53) = − .09, *p* = .52	r(53) = − .08, *p* = .58	r(53) = .05, *p* = 70	r(53) = − .10, *p* = .89

Spearman’s Rho was also used to assess whether scores on the outcome variables were associated with any of the predictors. The results showed a significant association between all variables. Further, the results showed a high but not perfect correlation between social support and belonging (r(58) = 0.81, *p* =  < 0.01), confirming that whilst there is some overlap, the social support scale and belongingness scale were measuring two distinct constructs. The results are presented in Table 5.

**Table 5:** Association between input and outcome variables

Variable	Belonging	Gambling Urges	Quality of Life
Social support	r(58) = .81, *p* = < .01	r(58) = − .72, *p* = < .01	r(58) = .69, *p* = < .01
Belonging		r(58) = − .77, *p* = < .01	r(58) = .71, *p* = < .01
Gambling urges			r(58) = − .81, *p* = < .01

**Fig. 1:**
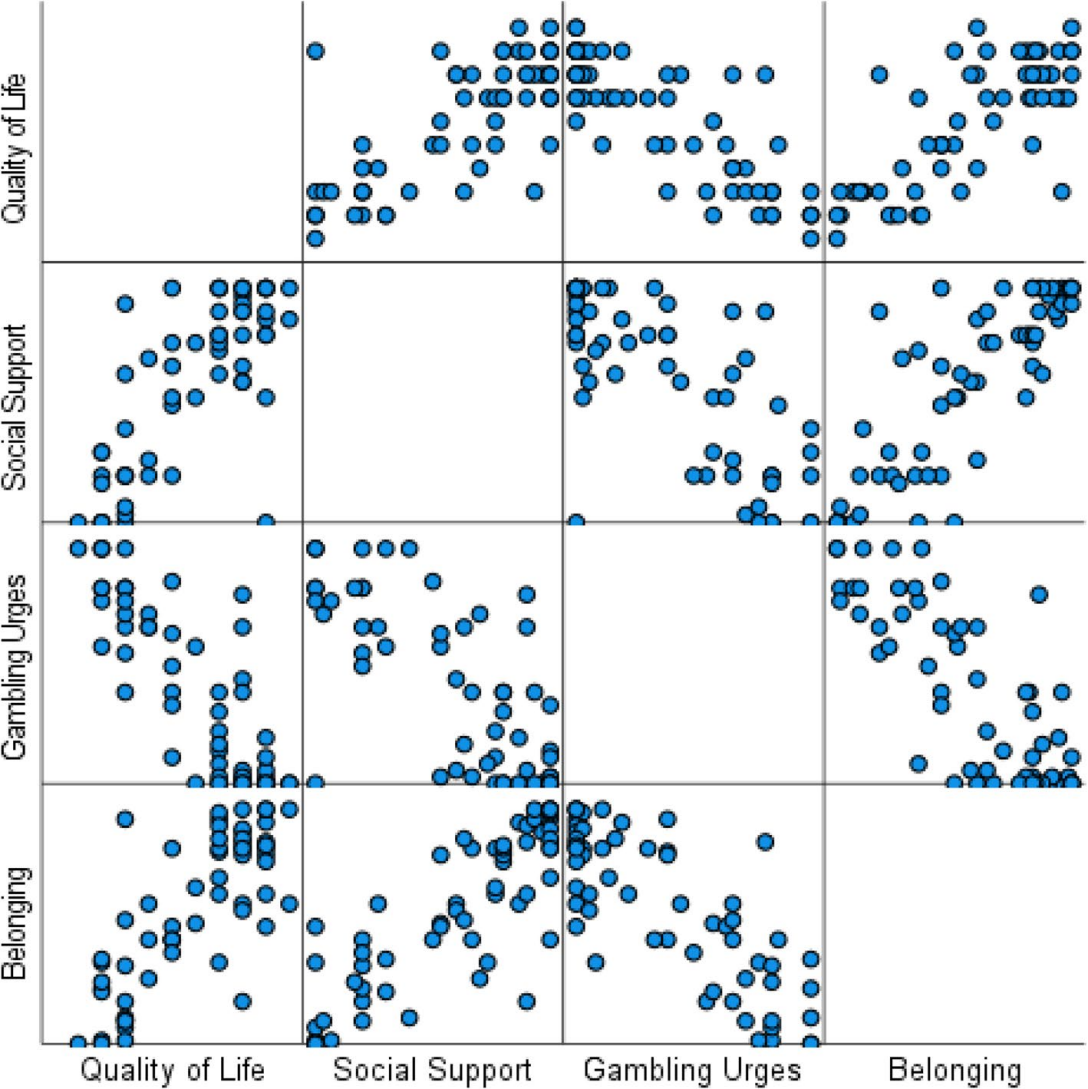
Scatterplot matrix of predictor and outcome variables

A summary of these results is presented in Fig. 1.

(v) The role of GA membership, social support and belonging in predicting addiction recovery

**Fig. 2:**
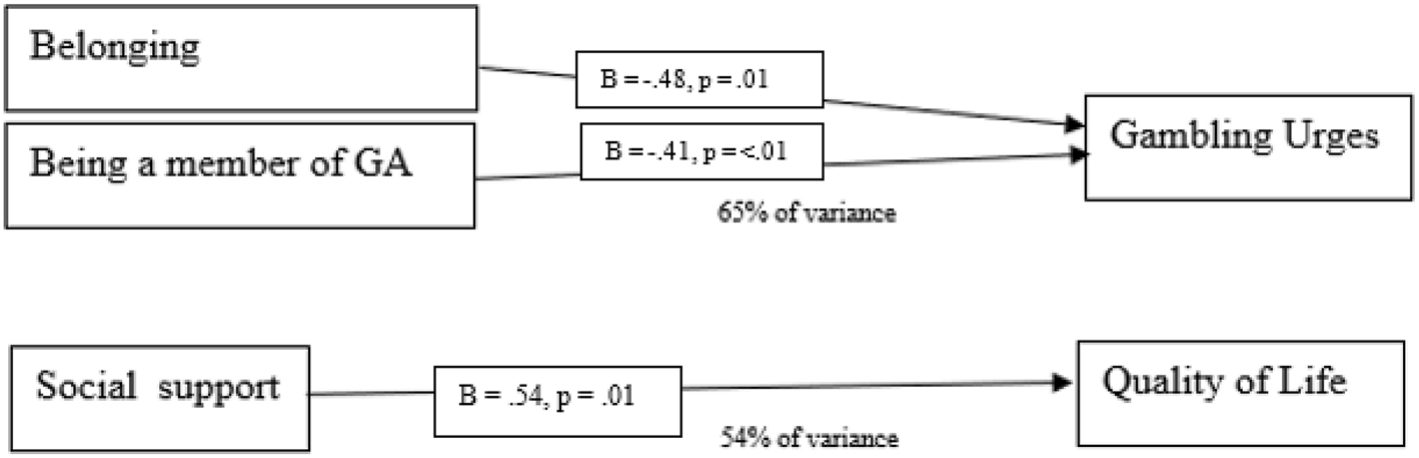
Summary of regression analysis results

Finally, a regression analysis was performed for each of the outcome variables with the significant demographic variables (membership to GA, duration of membership) and input variables (social support, belonging) as predictor variables. The results are presented in Table 6. A summary of the results is also presented in Fig. 2.

**Table 6:** Predictors of gambling addiction recovery (regression analysis)

Variable	Gambling urges	Quality of life
Social support	B = − .15, *p* = .40	B = .54, *p* = .01
Belonging	B = − .48, *p* = .01	B = .18, *p* = .37
GA membership	B = − .41, *p* = < .01	B = .168, *p* = .27
Duration of GA membership	B** = **− .15, *p* = 35	B = .138, *p* = .45
*R*^2^, final model	65%	55%

#### Gambling Urges

The results showed that feelings of belonging (*B* = *0.4*8, *SE* = 0.20,* p* = 0.01, and being a member of GA (*B* = − 0.41, *SE* = 0.70, *p* =  < 0.01) predicted gambling urges accounting for 65% of the variance (*F* = 10.74, *R*^2^ = 0.65,* p* =  < 0.01). This indicates that lower gambling urges were predicted by higher belonging, and being a member of GA. No role was found for social support (*B* = 0.15, *SE* = 0.29, *p* = 0.40) or duration of GA membership (*B* = 0.15, *SE* = 0.25, *p* = 0.35). These results are shown in Fig. 2.

#### Quality of Life

The results showed social support predicted quality of life (*B* = 0.54, *SE* = 0.28, *p* = 0.01), accounting for 54% of the variance (*F* = 7.47, *R2* = 0.55, *p* =  < 0.01) indicating that higher quality of life is predicted by higher social support. No role was found for belonging (*B* = 0.18, *SE* = 0.20, *p* = 0.37), GA membership (*B* = 0.168, *SE* = 0.67, *p* = 0.27), and duration of GA membership (*B* = 0.138, *SE* = 0.25, *p* = 0.45).

## Discussion

Whilst research indicates a role for both social support and belonging in addiction recovery, little is known about the role of these constructs in the recovery from problem gambling, and whether they relate to the effectiveness of mutual aid groups such as GA. The present study therefore aimed to explore the relationship between social support and belonging and to assess the role of demographics (including group membership of GA), social support and/or belongingness in predicting gambling addiction recovery in terms of both gambling urges and quality of life.

The results of this study first showed that, whilst there was significant overlap between social support and belongingness, they were still two discrete constructs. Therefore, whilst previous research has at times used the constructs interchangeably the results from the present study indicate that they are distinct. This is further illustrated by their differential impact on addiction recovery.

In terms of gambling urges, the results showed that lower gambling urges were predicted by higher feelings of belonging, and being a member of GA. No role was found for social support, gender, age, ethnicity, employment status, level of education or duration of GA membership. Hutchison et al. (2018) demonstrated giving social support, rather than receiving it, was linked to recovery-related outcomes, and that when individuals identify with a recovery group, they are more likely to give social support to that group. If belonging refers to “a unique and subjective experience that relates to a yearning for connection with others, the need for positive regard and the desire for interpersonal connection” (Rogers, 1951), it could be argued that identifying with the recovery group is akin to belongingness. In this context, the results of the present study align with those of Hutchison et al. (2018); feelings of belonging facilitate the provision of social support which in turn aids recovery. In this way, social support may still play a role in gambling-specific recovery outcomes, however the direction of this social support may have been overlooked in this study. Future researchers should explore the bi-directional nature of social support within mutual aid group settings further; perhaps this study could be replicated, adding a measure of provision of social support.

In contrast, quality of life was predicted by higher social support. No role was found for belonging, gender, age, ethnicity, employment status, level of education, membership to GA or duration of GA membership. These results support previous research (e.g. Best et al., 2016; Buckingham et al., 2013; Dingle et al., 2015) by demonstrating that feelings of belonging, and feelings of social support, both contribute to gambling addiction recovery. That membership to GA predicted a reduction in gambling urges but not an increase in quality of life could reflect the gambling-specific nature of GA which might specifically help with gambling urges, whereas social support might more broadly improve the lives of individuals in all aspects of their life.

Some studies have indicated that mutual aid groups function via several mechanisms simultaneously (Kelly et al., 2020; White et al., 2020). Penfold and Ogden, (2021; 2022 under review) found that a feeling of “connection” to a recovery group was an underlying mechanism behind its success; their qualitative studies suggested that this “connection” was an intricate combination of both belonging and social support. The results of the present study support the previous work of Penfold and Ogden, (2021; 2022 under review) as they suggest that both social support and belonging play a role in gambling addiction recovery, although these roles may be different, and that whilst belonging (and belonging to GA) predicts a reduction in gambling urges, it is social support that predicts quality of life.

In the context of these previous studies, an explanation of these results could be that individuals may use gambling to ‘fill a hole’ when they feel disconnected or feel they don’t belong; once they feel they belong somewhere that hole is ‘filled up’ and their gambling urges subside. This would also explain why attending GA specifically helps to reduce gambling urges, as they are remedied by the feeling of belonging GA gives them. It might also more broadly explain why the majority of gamblers never develop problems (Wardle et al., 2018), or why many people find their gambling problems subside without intervention (Slutske, 2006); these people may be protected by belonging. In contrast, social support may help resolve gambling problems in a more indirect or practical way, through improving people’s lives by giving them the resources and support they need to seek help. Finally, these novel findings demonstrate that more research in this area is required, as more research may help guide decisions about, and development of, treatment for problem gamblers in the future.

In a study comparing QoL across gambling, drug and alcohol addictions, Manning and colleagues (2012) found those with gambling addictions had lower QoL than those with alcohol addictions leading the authors to conclude that factors such as the breakdown of their familiar relations reduced their quality of life (Manning et al., 2012). In comparison, those with alcohol addictions were protected by the social support received from their familiar network. Petry and Weiss (2009) also demonstrated that social support plays an important role in moderating treatment outcomes, and enhancing social support may be an important aspect of effective treatments. Within the context of these previous studies, the implications of the current findings are significant. Firstly, recognising that social support and belonging are distinct constructs suggests that interventions and treatment approaches should address both factors separately.

These findings emphasise the importance of providing opportunities for individuals with gambling addiction to foster a sense of belonging within a supportive community, such as through participation in support groups like GA. Further, the results suggest that the sense of belonging, and connection experienced within these groups can be a powerful motivator and protective factor against relapse. In the context of previous literature, these results suggest that treatments could be developed which target specific areas of a person’s life such as repairing familial relationships to improve QoL. The implications also suggest that treatment approaches for gambling addiction should not solely rely on social support networks but should actively cultivate a sense of belonging. This might involve creating supportive environments, promoting peer-to-peer connections, and developing interventions that address the unique psychological and emotional needs associated with a sense of belonging. Indeed, though previous research in this area is scarce, Bickl and colleagues (2023) recently found similar results, demonstrating that strengthening social-emotional resources may be a promising strategy for mitigating or even preventing gambling-related problems.

Overall, these findings highlight the importance of addressing social and relational factors, particularly the sense of belonging, in designing effective interventions and support systems for individuals with gambling problems. By incorporating these elements, treatment approaches can enhance recovery outcomes and overall well-being for individuals struggling with gambling addiction.

## Limitations

There are some problems with the present study that need to be considered. There is a difficulty in studying mutual aid groups, both ethically and logistically. As membership to the groups requires a desire to be sober, one cannot easily measure gambling reduction directly, which is why this study used gambling urges and quality of life as a measure of gambling addiction recovery. This may have been a limitation however. The results showed that each of the input variables predicted a different outcome variable, though the outcome variables were assumed to be connected, i.e. it was expected that any effect shown would be shown for both measures, however this was not the case. This also highlights one of the broader issues with gambling addiction recovery research (and indeed more broadly, mental health recovery) – that of measurement, and how exactly one quantifies ‘recovery’. There have been compelling arguments made for a myriad of different ways to measure gambling addiction, including gambling symptoms (The Gambling Symptom Assessment Scale; Kim et al., 2009), urges (The Gambling Urges Scale; Raylu & Oei, 2004a, 2004b), addiction severity (Addiction Severity Index;(McLellan et al., 1980), by following up gambling treatment (Gambling Follow-up Scale; de Castro et al., 2005), gambling motives (Gambling Motives Questionnaire; Stewart & Zack, 2008)), problem gambling severity (Problem Gambling Severity Index; Ferris & Wynne, 2001), gambling related cognitions (Gambling Related Cognition Scale; Raylu & Oei, 2004b), gambling motivation (The Gambling Motivation Scale; Lafrenière et al., 2012), attitudes towards gambling (Attitudes Towards Gambling Scale; Canale et al., 2016) to name a few. There are also various screening tools. This list demonstrates the absence of a concrete underlying theory of gambling addiction, or of how that might be measured. The authors of this study chose the measures which were most applicable to the sample and nature of the study, however there may have been an oversight regarding whether these two measures could be used to measure the same thing.

Another limitation is that the study was cross-sectional in design, and data were collected almost exclusively through social media (primarily Instagram) which may have limited the sample (to only those using Instagram). Many participants also chose not to give any demographic information. As such, the data from these respondents is unknown and could not be included in the analysis. Furthermore, whilst every effort was made to recruit a diverse sample, the majority of participants (who did provide demographic information) were white males which could have limited the results. Future researchers should broaden recruitment methods, and aim to capture data from a more ethnically and gender diverse range of participants.

A final limitation is that all the data were self-report and are subject to well-known biases (e.g. memory or social desirability biases). To overcome such biases, further research with differing methodologies is required to answer the same questions (such as qualitative and/or observational). Given the present study is one of only a handful of studies focusing on GA in the UK, and the first to investigate and compare the roles of belonging and social support in gambling addiction recovery, future research should look to elaborate on the findings of this study, particularly focusing on the role of belonging and providing social support in gambling addiction recovery.

Despite these limitations, the results of this study, and the research which it is hoped it will generate, has the potential to contribute to addiction recovery and mutual aid group understanding, potentially changing the way treatment providers and other healthcare professionals tackle the issue.

## Conclusions

In conclusion, the results from the present study indicate that social support and belong, whilst inter-related, are discrete constructs and have a differential impact on gambling addiction recovery. In particular, whilst higher belonging, and being a member of Gamblers Anonymous were found to predict lower gambling urges, higher social support predicted better quality of life.

These results indicate firstly that social support and belonging should be considered as different constructs, and secondly the social process underpinning the effectiveness of Gamblers Anonymous is the sense of belonging it provides its members, rather than social support. Finally, these novel findings demonstrate that more research in this area is required, as more research may help guide decisions about, and development of, treatment for problem gamblers in the future.

## Data Availability

The datasets generated during and/or analysed during the current study are available from the corresponding author on reasonable request.
